# Chaperone Proteins: The Rising Players in Muscle Atrophy

**DOI:** 10.1002/jcsm.13659

**Published:** 2024-12-20

**Authors:** Davide Acquarone, Alessandro Bertero, Mara Brancaccio, Matteo Sorge

**Affiliations:** ^1^ Department of Molecular Biotechnology and Health Sciences University of Turin Turin Italy

**Keywords:** cachexia, chaperone proteins, heat shock proteins, muscle atrophy, proteostasis, sarcopenia

## Abstract

Despite significant progress in understanding the molecular aetiology of muscle atrophy, there is still a great need for new targets and drugs capable of counteracting muscle wasting. The role of an impaired proteostasis as the underlying causal mechanism of muscle atrophy is a well‐established concept. From the earliest work on muscle atrophy and the identification of the first atrogenes, the hyper‐activation of the proteolytic systems, such as autophagy and the ubiquitin proteasome system, has been recognized as the major driver of atrophy. However, the role of other key regulators of proteostasis, the chaperone proteins, has been largely overlooked. Chaperone proteins play a pivotal role in protein folding and in preventing the aggregation of misfolded proteins. Indeed, some chaperones, such as αB‐crystallin and Hsp25, are involved in compensatory responses aimed at counteracting protein aggregation during sarcopenia. Chaperones also regulate different intracellular signalling pathways crucial for atrogene expression and the control of protein catabolism, such as the AKT and NF‐kB pathways, which are regulated by Hsp70 and Hsp90. Furthermore, the downregulation of certain chaperones causes severe muscle wasting per se and experimental strategies aimed at preventing this downregulation have shown promising results in mitigating or reversing muscle atrophy. This highlights the therapeutic potential of targeting chaperones and confirms their crucial anti‐atrophic functions. In this review, we summarize the most relevant data showing the modulation and the causative role of chaperone proteins in different types of skeletal muscle atrophies.

## 1 Introduction

Skeletal muscle is a highly plastic and dynamic tissue that accounts for 40%–55% of the overall body weight. Skeletal muscles have several fundamental functions, being involved in posture, movement and breathing, but also serving as the primary reservoir of amino acids and glucose of our body.

Along with genetic diseases, other pathological conditions can cause a functional decline in muscles. Muscle atrophy is a clinical manifestation associated with various pathologies, such as heart failure, cancer cachexia, obesity, diabetes, injuries, as well as aging, bed rest, and inactivity, thus representing a co‐morbidity with a relevant medical burden [1]. Muscle atrophy consists in the loss of muscular mass and strength due to myofibre shrinkage. Its primary hallmark is an imbalance of proteostasis, characterized by a shift toward a hypercatabolic state, not adequately counterbalanced by protein anabolism. Indeed, pathological events activate a combination of signalling pathways, in the different atrophic conditions, resulting in specific proteomic and transcriptomic changes involving both protein catabolism and anabolism [2, 3]. The complexity of this process hampers the development of effective therapeutic strategies in muscle atrophy.

**FIGURE 1:**
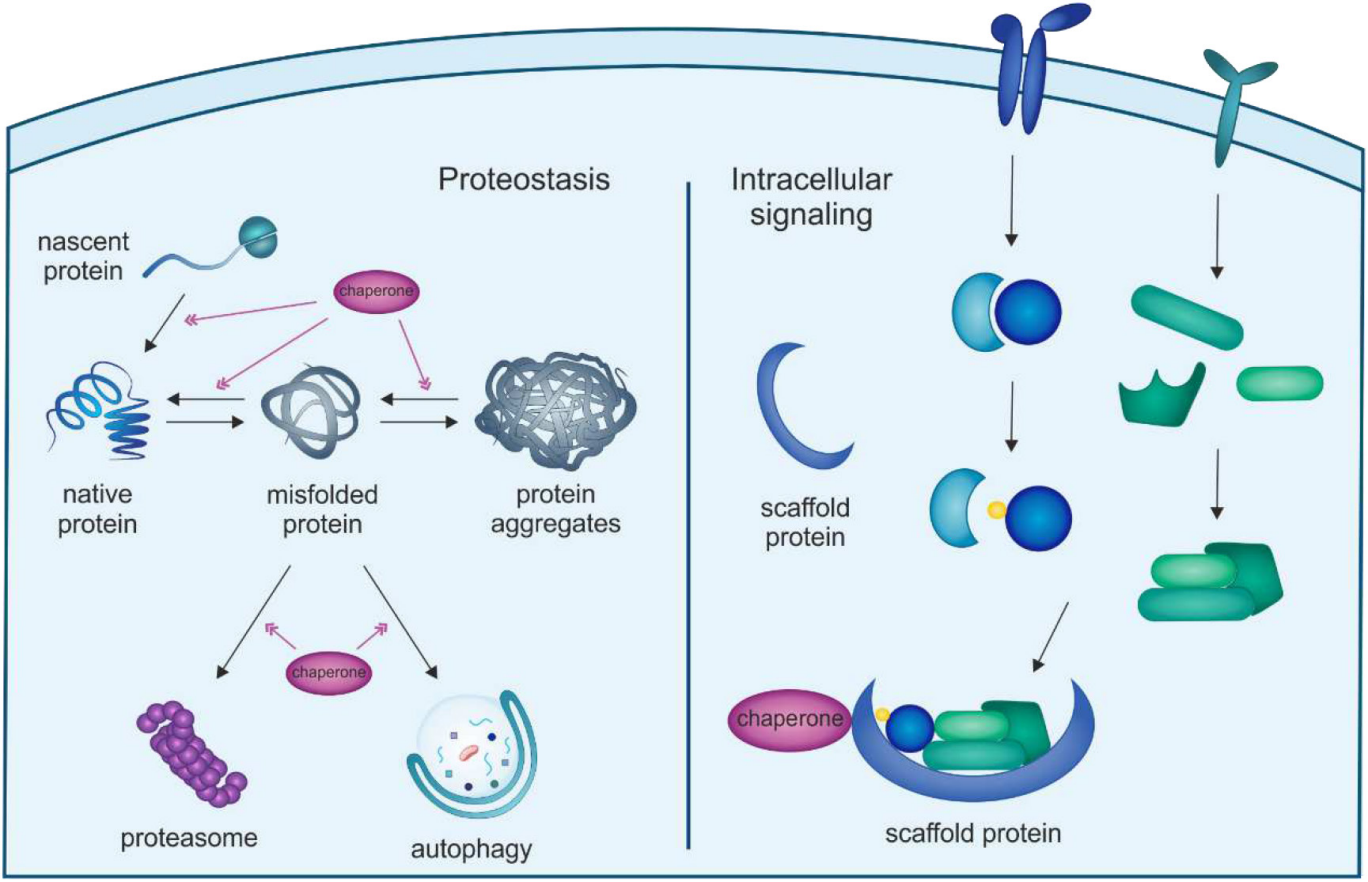
The dual role of chaperone proteins in cells. Chaperones regulate proteostasis at multiple layers. They fold nascent polypeptides, prevent accumulation of misfolded proteins and aggregation events by cooperating with the ubiquitin proteasome system and autophagy. Chaperones also coordinate intracellular signalling pathways by promoting the assembly of signalling protein complexes together with scaffold proteins.

Chaperone proteins are key regulators of cellular proteostasis [4]. They fold nascent proteins, re‐fold misfolded polypeptides, prevent protein aggregation and direct unfolded proteins to degradation through the ubiquitin proteasome system (UPS) and the autophagic system. Chaperones are also in charge of forming supramolecular complexes together with scaffold proteins, thereby facilitating and regulating different signalling pathways [5] (Figure 1). When cellular homeostasis is impaired by stress conditions, such as oxidative damage, hyperthermia and mechanical stress, chaperone proteins are upregulated as part of the so‐called heat shock response (HSR), a compensatory reaction aimed at restoring the physiological proteostasis [6] (Table 1). In muscles, chaperone proteins regulate the turnover of sarcomeric proteins, often unfolded by mechanical stretch, preserving muscle contractile function. This is in line with evidence that mutations in chaperone genes cause certain types of myopathies [7, 8]. Given the pivotal role of chaperones in proteostasis and signalling pathway regulation and the relevance of these processes in skeletal muscle atrophy, chaperones appear as potential key players in muscular atrophy that have been largely overlooked.

**TABLE 1:** Basic features of heat shock proteins cited within the text.

Gene symbol (approved as official by HGNC)	Protein names	Tissue distribution	Stress‐inducible
HSPB1	Hsp25; HSP27; HSP28	Ubiquitous	Yes
CRYAB	αB‐crystallin; HSPB5; CRYA2	Ubiquitous	Yes
HSPB6	Hsp20; PPP1R91	Ubiquitous	Yes
HSPB7	cvHsp; HspB7	Heart, skeletal muscle, adipose tissue	Yes
HSPD1	Hsp60; GroEL	Ubiquitous	Yes
HSPE1	Hsp10; GroES; CPN10	Ubiquitous	Yes
HSPA1A	Hsp70–1; Hsp72	Ubiquitous	Yes
HSPA1B	Hsp70–2; Hsp72	Ubiquitous	Yes
HSPA9	Grp75; mtHSP75	Ubiquitous	Yes
HSPA8	Hsc70; HSPA10	Ubiquitous	No
HSP90AA1	Hsp90	Ubiquitous	Yes
HSP90AB1	Hsp90B	Ubiquitous	Yes
HSP90B1	Grp94; GP96	Ubiquitous	Yes
ITGB1BP2	Melusin	Heart, skeletal muscle	Yes
STUB1	CHIP; Stub1	Ubiquitous	Yes

The aim of this review is to report the already known roles of chaperone proteins in the different types of skeletal muscle atrophy (Table 2), highlighting their pivotal role in the atrophic process and their potential as therapeutic targets.

**TABLE 2:** Summary of the known modulation of chaperone protein in the main type of muscle atrophy.

Gene name	Protein name	Disuse‐induced atrophy	Denervation‐induced atrophy	Sarcopenia	Cancer cachexia	Anti‐atrophic function experimentally demonstrated	References
HSPA1A, HSPA1B	Hsp70	Decrease protein level	Decrease protein levels	Controversial reports on Hsp70 modulation in aging Increase of eHsp72	Increase eHsp72	Disuse atrophy Ventilator‐induced diaphragm dysfunction	[9, 10, 11, 12, 13, 14, 15, 16, 17, 18, 19, 20, 21, 22, 23]
HSPA8	Hsc70	No modulation	N.A.^a^	Decrease protein level	N.A.^a^	—	[10, 14, 24, 25]
HSPA9	Grp75	Transient increase followed by downregulation	Decrease in protein level	Decrease in protein level	N.A.^a^	—	[26, 27, 28]
HSP90AA1, HSP90AB1	Hsp90	Decrease in protein and mRNA level	N.A.^a^	Decrease in protein level	Increase protein level in human Increase of eHsp90	—	[14, 23, 29, 30, 31, 32]
HSP90B1	Grp94	Decrease in protein level	N.A.^a^	N.A.^a^	N.A.^a^	Disuse induced atrophy	[33, 34, 35]
ITGB1BP2	Melusin	Decrease in protein level	N.A.^a^	N.A.^a^	N.A.^a^	Disuse induced atrophy	[36]
CRYAB	αB‐crystallin	Decrease in protein and mRNA level	Decrease protein level in slow twitch muscles	Increase protein level	N.A.^a^	—	[13, 14, 21, 37, 38, 39, 40, 41, 42]
HSPB7	HspB7	N.A.^a^	Controversial reports on HspB7 modulation after denervation	Controversial reports on HspB7 modulation in aging	N.A.^a^	—	[21, 25, 38, 40]
HSBP1	Hsp25	Decrease protein and mRNA level	Decrease protein level	Controversial reports on Hsp25 modulation in aging	N.A.^a^	Disuse induced atrophy	[13, 14, 18, 25, 40, 41, 43, 44]
HSPB6	Hsp20	Decrease protein level	Transient increase followed by downregulation	Controversial reports on Hsp20 modulation in aging	N.A.^a^	—	[13, 14, 21, 29, 45, 46, 47]
HSPD1	Hsp60	N.A.^a^	N.A.^a^	Controversial reports on Hsp60 modulation in aging	N.A.^a^	—	[18, 21, 25]
HSPE1	Hsp10	N.A.^a^	N.A.^a^	Decrease in protein level	N.A.^a^	Sarcopenia	[25, 48]
STUB1	CHIP	N.A.^a^	N.A.^a^	Increase protein level	N.A.^a^	—	[49]

^a^ Data not available.

## 2 Hsp70 Protein Family

### 2.1 Hsp70

Hsp70 refers to two proteins that differ only by two amino acidic residues, encoded by two different genes, *HSPA1A* and *HSPA1B*. Both are often named Hsp70 or Hsp72 due to the high grade of homology and their interchangeable function [50]. Hsp70 is a stress‐inducible chaperone, ubiquitously activated in response to physiological and pathological stimuli, such as intense exercise, reactive oxygen species (ROS), metabolic alterations and heat. Hsp70 controls protein folding and regulates several signalling pathways and cellular processes [S1].

In skeletal muscles, Hsp70 plays a key role in maintaining sarcomere structures and muscle integrity. Indeed, mice knockout for Hsp70 show a reduced myofibre cross‐sectional area (CSA) and increased extracellular matrix deposition between myofibres in baseline conditions [50]. In line, knockdown of Hsp70 in L6 rat myotubes leads to a significant reduction in their diameter, an increase in FoxO3 transcriptional activity, Atrogin‐1 expression and proteasome activity [51]. Other studies have also suggested a role for Hsp70 in muscle regeneration after injury. In particular, the overexpression of Hsp70 facilitates the regeneration and recovery from injuries induced by cardiotoxin injection or cryo‐lesions [50, 52].

The reduced expression of Hsp70 during muscle atrophy induced by disuse [9, 10, 11, 12, 13, 14, 15] and denervation [16] further suggests that this chaperone plays a role in muscle integrity and in the protection from muscle wasting. It has been demonstrated that low levels of Hsp70, together with heat shock cognate 70 (Hsc70), slow the rate of protein elongation and consequently decrease protein synthesis. Increasing evidence sustains that Hsp70 can bind to phosphorylated AKT (p‐AKT), preventing its dephosphorylation and sustaining active anabolic pathways in skeletal muscles [53, 54]. Of note, the overexpression of Hsp70 in myotubes protects from the reduction of p‐AKT during muscle atrophy induced by dexamethasone (DEX) [53, 54].

The decline of Hsp70 during muscle atrophy is further sustained by a positive feedback cycle. The reduction of Hsp70 induced by DEX results in an increase in nuclear translocation of the Glucocorticoid Receptor (GR), that, in turn, lead to the expression of miRNA‐1, a GR target [54]. miRNA‐1 is a myo‐miRNA promoting myogenesis, known to be downregulated during muscle hypertrophy [55]. Hsp70 owns a miRNA‐1 regulatory element in its 3’UTR, thus, the upregulation of miRNA‐1 further reduces Hsp70 expression, establishing a feedback loop [54]. Whether this molecular circuit is common among different types of muscle atrophy is still unclear.

Besides regulating protein synthesis, by supporting anabolic signalling and chaperoning protein synthesis, Hsp70 also modulates protein degradation. Various studies sustain that Hsp70 plays a negative regulatory role within two prominent pathways associated with muscle wasting: FoxO3a [11, 56] and NF‐κB [12]. Senf and co‐workers [11] demonstrated that the overexpression of Hsp70 prevents muscle wasting induced by unloading, promoting the phosphorylation of FoxO3a by AKT and the suppression of Atrogin‐1 and MuRF1 induction. Hsp70, by maintaining p‐AKT levels, contributes to AKT‐dependent phosphorylation and inhibition of FoxO3a. Nevertheless, experimental data indicate that Hsp70 prevents FoxO3a transcriptional activity also through an AKT‐independent and still unknown mechanism. Indeed, it has been demonstrated that Hsp70 overexpression hampers the transcriptional activity of a nonphosphorylatable FoxO3 mutant [56].

Hsp70 can also inhibit NF‐κB signalling activation. Indeed, in disuse‐induced skeletal muscle atrophy, Hsp70 overexpression upregulates the NF‐κB repressor IκB‐α [12].

Hsp70 exerts an anti‐atrophic function also in ventilator‐induced diaphragm dysfunction (VIDD), a common type of diaphragm atrophy experienced by patients undergoing prolonged mechanical ventilation, a frequent medical intervention during the COVID‐19 pandemic. AAV‐mediated Hsp70 overexpression was sufficient to prevent diaphragm atrophy in rats subjected to mechanical ventilation [17].

Contrasting evidence exist on the regulation of Hsp70 expression during sarcopenia. Chung et al. [18] reported a slight increase in Hsp70 content in the gastrocnemius of 29‐month‐old rats compared to 16‐month‐old ones, in contrast to different reports indicating a similar content in old and adult rats [19, 20] or a slight decrease in old rats [12, 21]. However, pharmacological induction of Hsp70 with 17‐(allylamino)‐17‐demethoxygeldanamycin (17‐AAG) or Hsp70 transgenic overexpression ameliorates the maximal force in old murine muscles and favours the recovery from force deficit caused by contraction‐induced injuries [57, 58]. However, both Hsp70 pharmacological induction and overexpression failed to protect from the sarcopenia‐induced decline of muscle CSA [20, 57].

Intriguingly, while intracellular Hsp70 has an anti‐atrophic effect in skeletal muscles, high levels of extracellular Hsp72 (eHsp72) are associated with skeletal muscle wasting. Given the positive correlation observed in a study on elderly individuals, eHsp72 serum levels may represent an effective biomarker of sarcopenia [22]. Indeed, a high level of eHsp72 is characteristic of the so‐called “inflammaging”, a low‐grade chronic inflammatory state peculiar to aging, while a low eHsp72 serum level has been associated with healthy aging and exceptional longevity [59]. The worth of this sarcopenia‐biomarker was directly supported by a study in which old, sarcopenic patients underwent 16 weeks of endurance exercise training, a widely known nonpharmacological approach proven to slow down sarcopenia onset [60]. The physical regime was able to drastically reduce eHsp72 serum levels concomitantly with a rise in lean body mass and muscle mass index of patients [60]. The rise of eHsp72 has also been reported in cancer cachexia‐induced skeletal muscle wasting. Cancer cells can secrete microvesicles containing Hsp70 and Hsp90, which trigger muscle atrophy and promote the onset of an inflammatory state [23]. Systemic administration of Hsp72 and Hsp90 in mice are able per se to induce muscle catabolism and atrophy through the activation of TLR4 signalling pathway in myofibres [23].

### 2.2 Hsc70

Heat shock cognate 70 (Hsc70) is a molecular chaperone member of the Hsp70 family, coded by the *HSPA8* gene, which share the 85% homology with Hsp70. Hsc70 is ubiquitously expressed in tissues and can represent up to 1% of the total cellular protein content. In cells, Hsc70 localizes predominantly in the cytoplasm, but it can also be present in the nucleus where it is involved in protein import [61]. Unlike Hsp70, Hsc70 is constitutively expressed and only mildly inducible upon stressful stimuli. Hsc70 functions vary from protein folding or degradation of misfolded proteins, to chaperone‐mediated autophagy and protein translocation [61].

In skeletal muscles, Hsc70 exerts a specific role in myofibrillogenesis. A supramolecular complex containing Hsc70, Hsp90 and Unc‐45b is responsible for the initial folding of newly synthesized myosins and for myofibril assembly [62]. A recent study has demonstrated that Hsc70 is upregulated during C2C12 myotube differentiation and the treatment with Hsc70‐selective siRNA blocks C2C12 differentiation as well as the expression of MyoD and MyoG, two key master genes of skeletal muscle cell fate commitment [63].

Differently from Hsp70, Hsc70 protein and mRNA levels remain constant during pathological muscle atrophy, in line with its essential housekeeping role [10, 14]. Nonetheless, rising evidence suggests that Hsc70 functions can be altered during skeletal muscle atrophy. Hsc70 is required to promote the binding of the GR with Hsp90, a crucial step for the proper function and maturation of GR [64]. Hsc70 acetylation on Lysine 128 hampers its interaction with the GR [65]. A class II non histone specific deacetylase, HDAC4, which has been correlated with muscle atrophy induction [66], mediates the deacetylation of Hsc70 [65]. This favours Hsc70 interaction with GR and leads to a higher responsiveness of GR to its ligands, such as DEX, contributing to the muscle wasting phenotype [65].

Hsc70 has a key role in cellular proteostasis, as it is central in protein sorting through three different types of autophagy in mammals, endosomal microautophagy, chaperone‐assisted selective autophagy (CASA) and chaperone‐mediated autophagy (CMA) [67, 68]. Hsc70 binds to cytosolic proteins and selectively delivers them to the endosomal membrane, where they can enter by means of vesicles generated at the surface by the ESCRT complex (endosomal microautophagy) or by means of a lysosomal membrane receptor called lysosome‐associated membrane protein type 2A (LAMP2A) (chaperone‐mediated autophagy). In CASA, Hsc70 participates in the selective degradation of ubiquitin‐positive protein aggregates, in cooperation with the co‐chaperones Bcl‐2 associated athanogene‐1 (BAG1) and 3 (BAG3) [67, 68]. Despite the well‐accepted alterations of macro‐autophagy in muscle atrophy [1], the role of CMA, endosomal micro‐autophagy and CASA is still poorly investigated. It is known that autophagic processes, such as CASA, are crucial for the maintenance of myofibrillar structures and Z disk turnover, and that their alterations can result in myopathies, muscle weakness and atrophy onset [67, 69]. The established role of Hsc70 in these processes suggests that the deregulation of this chaperone could impact on the autophagic flux and in muscle wasting. Accordingly, in *Drosophila*, downregulation of Hsc70–4 by RNAi results in the impairment of CASA and in muscle defects, phenocopying the downregulation of Starvin (the *Drosophila* ortholog of mammalian BAG3) mutants [70].

During aging Hsc70 is downregulated in muscles [24, 25] and this correlates with the decline in the autophagic flux and the accumulation of protein aggregates in myofibres, which are considered the main contributors to muscle performance deficits in elderly [71, 72]. In *Drosophila*, the induction of autophagy by the dFOXO transcription factor improves proteostasis during aging. Of note, dFOXO also increases chaperone protein expression [72].

### 2.3 Grp75

Glucose‐related protein 75 (Grp75), also known as mitochondrial heat shock protein 70 (mtHsp70) or mortalin, is a 75 kDa heat‐uninducible member of the Hsp70 family, encoded by the gene *HSPA9B*. This chaperone mainly localizes within mitochondria, although it is also present in other cellular compartments, like the endoplasmic reticulum [73]. Grp75 plays many different housekeeping functions ranging from the regulation of mitochondrial protein import and intracellular Ca^2+^ homeostasis to proteasomal degradation, control of cell proliferation and differentiation, and antigen presentation in innate immunity [73]. Grp75 expression is not upregulated by heat shock, but it is mildly sensitive to other stressful stimuli, such as glucose deprivation, ionizing radiation and caloric restriction. The increase of Grp75 exerts a cytoprotective function by delaying apoptosis and promoting the cell antioxidant response [73] [S2].

In both human and rodent skeletal muscles, Grp75 exhibits an initial transient increase during the first days following unloading then starts to decline as muscle atrophy appears and progresses [26]. ROS levels rapidly increase during muscle wasting, triggering the activation of various pro‐atrophic drivers, such as FoxOs [74]. We can, thus, suppose that the initial upregulation of Grp75 may represent a physiological antioxidant response to mitigate the rise of ROS and prevent skeletal muscle wasting, though clear demonstrations are required.

In denervation induced muscle atrophy, Grp75 declines together with the mitochondrial translocase Tim23 and Tom20, resulting in a reduced mitochondrial protein import [27]. This reduction impacts on the mitochondrial network status fostering the mitochondrial dysfunction occurring with muscle atrophy [27].

Grp75 is also involved in the regulation of mitochondrial Ca^2+^ uptake from the sarcoplasmic reticulum (SR), which occurs at the level of the mitochondria associated membrane (MAM), a platform for inter organelle communication [75]. Grp75 tethers the N‐terminal domain of inositol 1,4,5‐triphosphate receptor (IP_3_R,) to the voltage dependent anion channel 1 (VDAC1) at the mitochondrial membrane, thus forming and stabilizing the macromolecular complex regulating mitochondrial‐SR Ca^2+^ transport. This is a critical cellular process that regulates mitochondrial function and energetic production and it has been hypothesized that the disruption of this process may contribute to the muscle dysfunction occurring in neurodegenerative diseases, Duchenne muscular dystrophy and aging [75].

Indeed, during aging there is a gradual reduction in the tethering between the sarcoplasmic reticulum and mitochondria, leading to a decrease in mitochondrial calcium uptake, excessive generation of ROS and consequent reduced myofibre function and muscle performance [76]. Intriguingly, sarcopenic muscles display a lower level of Grp75, together with VDAC1, IP_3_R and Mitofusin2 than young muscles, and this correlates to severe alterations in calcium homeostasis and ER stress, tubular aggregate formation and cell death, all factors reducing muscle performance in old mice [28].

Thus, given the multifaceted role of Grp75 in ensuring mitochondrial health, its reduction during atrophy may be responsible for the decrease in mitochondrial performance and muscle functions.

## 3 Hsp90 Protein Family

### 3.1 Hsp90

Hsp90 is an ATP‐binding chaperone protein conserved from prokaryotes to humans. The mammalian genome contains two genes encoding two highly homologous isoforms: the inducible Hsp90α and the constitutive Hsp90β. Hsp90 is highly expressed in cells, representing up to 2% of the total protein content and its level can rise, under stress conditions, up to 4%–6% [77]. Hsp90 works in cells as a dimer and forms a plethora of complexes with different substrates, called client proteins. It interacts with more than 40 co‐chaperones to exert several functions, from protein folding to signalling regulation. Given its essential functions, alterations in Hsp90 expression levels or activity are linked to many pathological conditions, from cancer growth and progression to neurological diseases [77] [S3].

In skeletal muscles, Hsp90 is required for the correct formation and organization of sarcomeres. Hsp90 binds and mediates the proper folding of myosin, together with Hsc70 and the co‐chaperone Unc45b. Studies in *C. elegans* and *Zebrafish* have shown that Unc45 targets unfolded myosin to Hsp90 and Hsc70, promoting the refolding of the myosin motor domains [78]. Inhibition of Hsp90 blocks myofibril assembly and leads to the accumulation of myosin folding intermediates [62]. Hsp90 regulates also myosin heavy chain (MyHC) gene expression, thereby influencing the overall myosin content and the myosin replacement rate in sarcomeres [79].

Differentiated myofibres express only the Hsp90β isoform, while Hsp90α gene is switched off during the differentiation process. Intriguingly, the sustained expression of Hsp90α hampers myotube formation, suggesting that this isoform switch is crucial for the muscular tissue development, even though the specific functional differences between the two cytosolic isoforms of Hsp90 in muscle fibres have yet to be clarified [80]. In skeletal muscle atrophy Hsp90 regulation differs according to the type of muscle atrophy. During unloading, Hsp90 protein and mRNA levels are significantly downregulated as early as 3 days after unloading [14, 29, 30] and restored after reloading [14, 29].

An excessive glucocorticoid stimulation causes a reduction of Hsp90 in muscles [81], this can sound counterintuitive, given the well‐known role of Hsp90 in steroid hormone signalling. Hsp90 interacts with the conformational labile ligand‐binding domain (LBD) of glucocorticoid receptors (GRs), holding it in a conformation prone to bind its ligands [64]. Therefore, reducing the presence of Hsp90 may potentially weaken the overall GR signalling. It is conceivable that following an initial role of Hsp90 in promoting the GR signalling cascade, the activation of specific gene programs might render Hsp90 dispensable. However, the biological mechanisms responsible for downregulating Hsp90, while keeping GRs active, remain to be fully defined.

Sarcopenia is associated with a severe downregulation of Hsp90β in skeletal muscles [82]. In aged mice, Hsp90β reduction results in the lower activity of the E3 ligase MDM2, a negative regulator of p53, and a consequent activation of the p53‐p21 axis. Reduced Hsp90 activity can enhance age‐related physiological impairment of muscles through p53 stabilization [82], as proved by the increased muscular senescence in mice treated with the Hsp90 inhibitor 17‐AAG [82]. Hsp90 also regulates AKT kinase, which is known to modulate myotubes differentiation, survival and hypertrophy [1]. Hsp90β binds to p‐AKT through its middle domain, stabilizing and protecting AKT phosphorylation from the action of the PP2A phosphatase [83]. Indeed, the inhibition of AKT‐Hsp90 interaction results in the dephosphorylation of AKT [83], while a long‐term inhibition of Hsp90 with geldanamycin results in the progressive depletion of total AKT, due to the inhibition of its folding and promotion of its degradation [84]. Therefore, the reduction of Hsp90 in skeletal muscles, impacting on AKT, can weaken the IGF‐1/AKT/mTOR signalling pathway, unbalancing proteostasis toward catabolism and protein degradation. Moreover, AKT phosphorylates and inhibits FoxO transcription factors, thus blocking their nuclear import and the consequent expression of pro‐atrophic genes, such as Atrogin‐1 and MuRF1 [1]. Intriguingly, treatment of DEX‐induced atrophic L6 myotubes with mountain ginseng resulted in an increased Hsp90 protein level, concomitantly with a dose‐dependent rise of p‐AKT, downregulation of FoxO3a and FoxO1 and inhibition of atrophy [81]. Even if in this study a stated causative link is missing, the correlation between Hsp90 induction and the reduced muscle wasting suggests a possible therapeutic role for Hsp90.

The mechanism by which Hsp90 is downregulated during skeletal muscle atrophy is largely unknown. The only players known to reduce Hsp90β protein content both in nonstriated and striated tissues are calpains, calcium‐dependent thiol proteases widely upregulated during unloading‐induced muscle wasting [85].

In skeletal muscle atrophy induced by cancer cachexia, Hsp90 is upregulated. Niu and colleagues [31] observed that abdominal muscles of cachectic cancer patients were characterized by an increased expression of Hsp90. This rise is associated with the increased expression of Myostatin, Atrogin‐1, MuRF1 and the enhanced activation of STAT3. Hsp90 interacts with phosphorylated STAT3 via its N‐terminal region [86], protecting STAT3 from the action of phosphatases, such as SHP‐1 [S4]. An increased interaction between Hsp90 and STAT3 has been observed in muscles of human cachectic patients [31], as well as in in vivo and in vitro models of cachexia‐induced atrophy. In myotubes in vitro, the silencing of Hsp90 or STAT3 protects from the atrophy induced by C26 cancer cell conditional media (C26‐CM) and decreases the expression of Atrogin‐1, Myostatin and MuRF1. The treatment of C2C12 myotubes with C26‐CM added with the Hsp90 inhibitor 17‐DMAG hampers STAT3‐Hsp90 interaction, reduces STAT3 activation and protects myotubes from atrophy. 17‐DMAG seems to improve muscle conditions also in several cachexia in vivo models even though in some cases it was not clear whether this was a consequence of the reduced tumour growth induced by Hsp90 inhibition [31]. These results indicate that Hsp90, through its interaction with STAT3, can promote muscle atrophy induced by cancer cachexia.

The reason for this contrasting role of Hsp90 in different types of atrophy depends on the fact that Hsp90 sustains various intracellular signalling pathways, some of which are involved in promoting muscle trophism, as the AKT pathway, while others are involved in activating atrogenes expression and unleashing muscle atrophy, as in case of STAT3 signalling. Depending on the pathways involved in triggering a specific type of muscle atrophy, Hsp90 may play a pro‐trophic or pro‐atrophic role, or even a mixture of the two.

It should be noted that Hsp90 modulates also others signalling pathways involved in muscle wasting, such as the nNOS and the NF‐κB pathways [74, 87], however to date, there are no indication for a direct contribution of Hsp90 in regulating atrophy through these signals.

During stress, some chaperone proteins are actively released in the extracellular milieu, where they exert biological activities by binding cell surface receptors and/or modulating extracellular client activity [88]. It has been discovered that extracellular Hsp90 (eHsp90) has a crucial role in promoting skeletal muscle wasting. In patients with pancreatic, lung or colon‐rectal cancer, at high risk of developing cachexia, elevated levels of Hsp90 and Hsp70 were detected in the serum, positively correlated with clinical stage, pathological tumour grade and mortality [32, 89, 90]. In line, ELISA assays performed on the conditioned medium from cachectic tumour cells (LLC, C26 and H1299) and on the serum of cachectic tumour bearing mice (LLC tumour bearing mice and Apc^min/+^ mice) have revealed a significant increase in eHsp90α and eHsp70 levels compared to noncachectic controls [23]. The administration of neutralizing antibodies against these chaperones completely abrogates muscle mass decrease, grip strength reduction and upregulation of atrophic markers, such as Atrogin‐1, UBR2 and LC3‐II, in different mouse models of cancer cachexia, without any effects on the tumour growth. In line, the exogenous administration of endotoxin‐free recombinant Hsp70 and Hsp90α in healthy mice promotes muscle wasting and recapitulates the atrophic phenotype [23]. eHsp90α and eHsp70 promote muscle catabolism by binding Toll‐like receptor 4 (TLR4) on the myofibre membrane, leading to the activation of the p38‐MAPK/C/EBPβ signalling pathway and autophagic flux and UPS activity [23, 91, 92]. eHsp90 and eHsp70‐mediated activation of TLR4 in immune cells also contribute to establish a systemic inflammation state during cachexia. Indeed, the impaired secretion of Hsp90 and Hsp70 in mice lacking Rab27 and injected with LLC cancer cells led to a significant reduction of TNFα and IL‐6 serum levels [23].

### 3.2 Grp94

Glucose‐regulated protein 94 (Grp94), also known as endoplasmin, is the most abundant glycoprotein in the endoplasmic reticulum (ER), originally discovered as a glucose sensor induced by glucose depletion [S3]. Grp94, encoded by the *HSP90B1* gene, is highly conserved in vertebrates and shares 50% homology with cytoplasmic Hsp90. Grp94 localization in the ER is mediated by an ER localization sequence of 21 amino acids in the N‐terminal region of the protein, cleaved upon entry into the ER lumen, and a C‐terminal ER retention sequence KDEL [93]. In certain tumour cell lines and in C2C12 myotubes, Grp94 has been also detected on the plasma membrane, although how Grp94 overcomes ER retention is still debated [S5, S6]. In the ER, Grp94 regulates the proper folding and maturation of a highly selective client list of secretory and membrane proteins, such as the folding intermediate of immunoglobulin light and heavy chains and IGF‐I/II [93]. Muscle specific Grp94 knockout results in ER‐associated protein degradation (ERAD) of IGF intermediates and, consequently, to smaller muscle and body size [94, 95]. Grp94 also possesses calcium‐binding properties, contributing to the overall calcium homeostasis [96] and, together with BiP/Grp78, is a key player of the unfolded protein response (UPR) during ER stress [S3].

In the context of skeletal muscle atrophy induced by disuse, Grp94 protein level is downregulated as early as 2 days after unloading [33, 34, 35]. Genetic and pharmacological approaches aimed to upregulate Grp94 resulted in partial protection from unloading‐induced muscle wasting [33, 34]. Myofibre transfection with Grp94 partially abrogated CSA decrease and the rise in protein carbonylation induced by hindlimb unloading [34], sustaining a possible antioxidant role of this chaperone in skeletal muscles. Of note, only myofibres transfected with Grp94 were protected from CSA decline, while not transfected myofibres retained the atrophic phenotype. This suggests that the Grp94 anti‐atrophic mechanism is cell‐autonomous and not dependent on IGF‐I release, which would have activated a paracrine positive effect also in not transfected fibres.

Curcumin intraperitoneal administration is one of the pharmacological treatments that partially counteracts skeletal muscle wasting, by maintaining muscle mass and strength and by antagonizing the rise in protein carbonylation [33]. Despite all the possible effectors of curcumin, it has been determined that curcumin exerts its action by mediating Grp94 rise, because the combined transfection of an antisense Grp94 cDNA completely hampers curcumin effects on skeletal muscles [33]. In physiological conditions, Grp94 promotes the targeting of nNOS to the sarcolemma where it binds to the DGC complex [97]. The loss of Grp94 drives disuse‐induced skeletal muscle atrophy by reducing nNOS levels at the sarcolemma and promoting its translocation to the sarcoplasm where it generates oxidative and nitrosative stress and activates FoxOs [35, 74, 87, 98]. Forced expression of Grp94 protects, via nNOS, from disuse‐induced muscle atrophy [33, 34]. How Grp94, which is mainly an ER luminal protein, maintains nNOS to the sarcolemma possibly depend on the ability of Grp94 to assume a transmembrane localization [S5].

## 4 Other Chaperone Proteins

### 4.1 Melusin

Melusin is a small chaperone protein encoded by the *ITGB1BP2* gene, exclusively expressed in striated muscle tissues. Melusin is one of the two members of the CHORD‐containing protein family encoded by the vertebrate genome [5]. Melusin is constituted by two CHORD domains capable of chelating zinc anions, together with a CS domain (CHORD‐containing protein and Sgt1 domain), structurally resembling the α crystallin domain of the p23 protein family, and a region enriched in aspartic and glutamic residues capable of binding Ca^2+^ [99]. Melusin cDNA was firstly discovered for its ability to bind the intracellular region of integrin β1 [S7], a component of costameres, which are supramolecular complexes connecting sarcomeres to the plasma membrane and the extracellular matrix responsible for the transduction of mechanical signals [74].

Unlike other chaperones, Melusin expression is not upregulated by heat stress, but by an increase in mechanical stretch. In the heart, Melusin organizes a supramolecular complex responsible for transducing mechanical signals [5]. It interacts with various signalling molecules, including the Focal adhesion kinase (FAK), the MAPK scaffold protein IQGAP1, mitogen‐activated protein kinases c‐RAF, MEK1/2, and ERK1/2, and the phosphoinositide 3‐kinase (PI3K) [5], likely regulating their assembly into a signalosome that activates hypertrophic and survival responses in the heart. Under pressure overload, Melusin triggers the activation of ERK1/2 and PI3K/AKT signalling cascades, promoting cardiomyocyte survival and compensatory hypertrophic remodelling of the myocardium, preserving cardiac contractility [5] [S8]. Melusin null mice fail to develop a compensatory hypertrophic response to pressure overload and rapidly progress to dilated cardiomyopathy with left ventricle dilation, chamber wall thinning, and impaired contractility [S8].

In skeletal muscles, Melusin starts to be expressed in mouse embryo limbs at 15 days of gestation, its expression peaks in newborn muscles and persists in adult skeletal muscles, where Melusin localizes at costameres, together with β1 integrin [S7]. Unloading conditions, as experienced in bedridden patients or during immobilization, impact costamere organization and Melusin expression [36, 74]. Hindlimb unloading results in a decreased Melusin protein level in the rat soleus muscle, as early as 6 h after suspension. This downregulation precedes the onset of muscle atrophy, which becomes evident only 2 days after unloading [36]. Analysis on human vastus lateralis biopsies reveals decreased Melusin protein levels already at 1 week of bed immobilization, when muscle atrophy is still undetectable [26, 35, 36].

AAV‐based gene therapy approach to counteract Melusin decline significantly protects against CSA reduction and Atrogin‐1 and MuRF1 mRNA rise, as well as improving muscle strength, as indicated by an increased tetanic and twitch tension [36]. Overall, these findings pinpoint Melusin as a key player in the onset of unloading‐induced muscle atrophy, even though additional studies are needed to define its downstream effectors and their mechanism of action.

Intriguingly, while Melusin protein level decreases already 6 h after unloading, its mRNA starts to decline only after 4 days, suggesting that Melusin drop depends on post‐transcriptional mechanisms [36]. Recent evidence suggests that Melusin is a substrate of the UBR4 ubiquitin ligase [100], which directly interacts and ubiquitinates Melusin. However, while UBR4 muscle‐specific knockout results in the hypertrophy of myofibres but not in muscle force increase [100, 101], Melusin‐based gene therapy both protects from atrophy and increases muscle contractility and strength [36].

### 4.2 Hsp60 and Hsp10

Hsp60 and Hsp10 are two chaperone proteins encoded by the nuclear genome, translated in the cytoplasm [S9] and translocated in the mitochondria. Hsp60 shares around 50% identity with the *E. coli* chaperonin GroEL. As GroEL, Hsp60 can assemble into double‐heptameric rings and Hsp10 associates on the top forming the Hsp60/10 chaperonin complex. This complex is responsible for folding proteins transported into the mitochondria, refolding denatured proteins and preventing the aggregation of denatured proteins in the mitochondrial matrix. Client polypeptides bind to the apical domain of the complex and the further binding of ATP induces a structural rearrangement that encapsulates the peptide into the HAP60 cavity and favours the association of Hsp10. ATP hydrolysis promotes the folding and the release of the client polypeptide together with the dissociation of HSP10 [S9].

The role of Hsp60 during muscle wasting has been primarily investigated in sarcopenia, obtaining contrasting results. Increased Hsp60 levels in the gastrocnemius of old rats have been correlated to apoptosis contributing to the increased muscle degeneration in old rats [18]. Other studies identified a downregulation of both Hsp60 and Hsp10 in both old rodents and humans, in line with a general decline of proteostasis occurring with aging [21, 25]. Hsp10‐overexpressing transgenic mice were protected from both CSA reduction and the loss of maximal tetanic force occurring during sarcopenia [48]. Hsp10 overexpression prevents the accumulation of oxidized mitochondrial proteins, a process that typically occurs in aged muscles.

### 4.3 Small Heat Shock Proteins

Small heat shock proteins (sHSPs) are a highly conserved protein family expressed across Archaea, Bacteria, and Eukarya. sHSPs function as ATP‐independent chaperones that bind misfolded proteins through hydrophobic residues to prevent aggregation events. Misfolded proteins, stabilized in a native‐like conformation by sHSPs, are then refolded by ATP‐dependent chaperones [S10]. sHSPs can also favour the degradation of aggregated proteins in cooperation with both the UPS and the autophagic machinery, playing a role in protein triage [S11]. sHSPs are characterized by an α‐crystallin domain (ACD) of 80–90 amino acids, forming an IgG‐like β sandwich structure [S10]. The ACD is necessary for the ability of sHSPs to oligomerize and form polydisperse complexes, that exert different functions inside cells from signal transduction to antioxidant and anti‐apoptotic responses and cytoskeletal stabilization during stress [S11, S12]. Most sHSPs are ubiquitously expressed (Table 1) and some of them, as αB‐crystallin, Hsp25 and HspB7, are highly expressed in the heart and skeletal muscles [102, 103]. In skeletal muscles, sHSPs are crucial for differentiation, tissue homeostasis and muscle integrity. Genetic mutations in sHSP genes, like αB‐crystallin, are linked to myopathies and muscle dysfunction [7, 104] and Hsp25 knockout in mice impairs muscle contraction and increases fatigue, highlighting the essential role of sHSPs in muscle function [105].

The regulation of sHSPs during skeletal muscle atrophy varies with the type of atrophy. Most sHSPs are downregulated during muscle atrophy induced by disuse (Table 2), including Hsp20, αB‐crystallin and Hsp25 in both rodents and humans [13, 14, 37, 45, 106].

In muscle disuse, reduced expression of αB‐crystallin is observed in slow‐twitch muscles, which are particularly susceptible to unloading‐induced atrophy, while an increased expression is detected in fast‐twitch muscles [37, 38, 39, 106], which are more resistant [107]. Whether the higher content of αB‐crystallin is responsible for the myofibre type resistance to atrophy is still to be defined. Hsp25 reduction in muscle disuse [14], correlates with an increase in oxidative stress, myonuclear apoptosis and proteolysis through UPR system and calpains [15]. The overexpression of Hsp25 prevents disuse‐induced skeletal muscle atrophy by blocking NF‐κB activity and atrogene expression [43].

During denervation‐induced muscle atrophy, Hsp20, αB‐crystallin and Hsp25 significantly decrease [37, 40, 44, 46, 106]. In particular, Hsp25 decline is less pronounced in muscles with residual nervous stimulation or residual passive stretch [40, 44]. The decrease in Hsp25 results in activation of caspase 3, which accelerates muscle contractile protein breakdown and atrophy [108].

In sarcopenia, αB‐crystallin is upregulated in both rodents and humans, possibly as a compensatory response to counteract damage and aggregation of contractile proteins [21, 41, 42, 47]. In particular, phosphorylated αB‐crystallin accumulates into insoluble protein fractions and this may represent an attempt to refold or to promote degradation of misfolded proteins [109], even if clear data are still lacking. Conversely, the regulation for most of other sHSPs, like Hsp25, is controversial, with studies reporting both decreased and increased expression levels in aged individuals (Table 2) [18, 25, 41, 110]. This may depend on the fact that, from the one hand, chaperone protein expression physiologically decreases during aging, and on the other hand, the accumulation of denatured proteins may potentiate their transcription, generating expression waves.

### 4.4 CHIP/STUB1

The carboxyl terminus of Hsp70‐interacting protein (CHIP, also known as STUB1) is a ubiquitin ligase that participates in the cellular protein quality control acting as a co‐chaperone of Hsc70, Hsp70 and Hsp90 [S13] [111]. CHIP associates with chaperones through its N‐terminal domain, while the C‐terminal domain acts as an E3 ligase that ubiquitinates the unfolded chaperone substrates, directing them to the autophagic or proteasomal degradation pathways [112, 113]. CHIP also modulates the expression of heat shock proteins, by controlling the transcriptional activity of heat shock factor 1 [114], and it induces the degradation of Hsp70 once the cellular stress response eases [115].

In the gastrocnemius of aged rats, as well as in models of muscular aging induced by silencing the ubiquitin ligase UBR4 or the proteasome component Regulatory particle triple‐A ATPase 3 (Rpt3), CHIP expression is upregulated as an adaptive response to promote the degradation of misfolded proteins [49, 101, 116].

Knocking out CHIP in *C. elegans* , *Drosophila* and mice leads to reduced life span and premature aging due to a disruption of cellular protein quality control and the decline of proteasome activity [113, 117]. In addition, CHIP‐deficient mice show reduced muscle size already at 3 months of age, compared to their wild‐type counterparts [113]. Experiments performed in *C. elegans* and *Drosophila* show that, during aging, CHIP is redirected to degrade accumulated damaged proteins, which reduce its ability to degrade the insulin receptor, thus boosting insulin signalling and promoting lifespan reduction [117].

## 5 Concluding Remarks

Muscle function is strictly dependent on the proper sarcomere organization, guaranteed by an effective and well‐programmed proteostatic network. Proteostasis is orchestrated by the coordinated action of three main components: chaperone proteins, autophagy and ubiquitin proteasome system, alias the triad of proteostasis. This coordinated network rapidly adapts to a large variety of stimuli; however, chronic stressors, like mechanical strain, oxidative damage, nutritional and energetic imbalance and inflammation, can overwhelm this ability, leading to proteostasis imbalance and impaired cellular function.

Muscle atrophy is activated in response to diverse events, as inflammation, disuse, absence of nerve signals or glucocorticoids stimulation. These stimuli activate various combinations of signal transduction pathways in muscle fibres that converge on the inhibition of protein synthesis and the promotion of protein degradation. Finding a successful approach to block these multiple signalling pathways, which can vary in response to different stimuli, has proven to be challenging. Chaperone proteins regulate protein synthesis and folding, drive protein ubiquitination and are involved in autophagy and, in the meantime, modulate signal transduction efficacy and integration among different pathways. This makes the chaperone system at the heart of the atrophic process. While the pivotal role of UPS and autophagy in inducing atrophy has been fully recognized as their potential values as therapeutic targets to counteract muscle loss, the relevance of chaperone proteins, the third component of the proteostasis triad, has been less investigated. Besides their direct role in skeletal muscle atrophy, heat shock proteins might exert systemic effects. Landmark studies in *Drosophila* and *C. elegans* [118, 119, 120] have shown that proteostasis perturbations in tissues such as skeletal muscles can trigger adaptive, non‐cell‐autonomous responses in other tissues. This cross‐organ response is mediated by myokines [119] and extracellular chaperones [118] and plays a critical role in sustaining multi‐organ proteostasis and resilience to stress. This opens up the fascinating hypothesis that proteostasis alterations in muscles, induced by aging and cachexia or disuse, can impact systemically in mammals as well. (Table 3).

**TABLE 3:** Open questions for future research on chaperone proteins in muscle atrophy.

Background	Unsolved questions
The reduction of several chaperone proteins has a causative role in inducing muscle atrophy.	What are the mechanisms responsible for the reduced expression of these chaperones?
While most chaperone proteins are decreased during atrophy, others are increased, likely as a compensatory response.	Can these chaperones be exploited as potential biomarkers?
Most of the studies investigate the role of single chaperones during muscle atrophy.	How the different types of atrophy shape the muscle chaperome? Does this impact on the composition and function of chaperone supramolecular complexes?
eHSPs released from the tumour cause muscle atrophy in mouse models of cancer cachexia.	Do extracellular heat shock proteins act as systemic signalling molecules inducing atrophy in other pathologies?
In not vertebrates, a trans‐cellular, cross‐organ signalling links proteostasis in different tissues.	Can proteostatic perturbations in muscle influence distal organs also in mammals?
Heat shock proteins are pivotal to muscle mass maintenance.	Which are the most promising strategies for modulating heat shock proteins to treat muscle atrophy?

**FIGURE 2:**
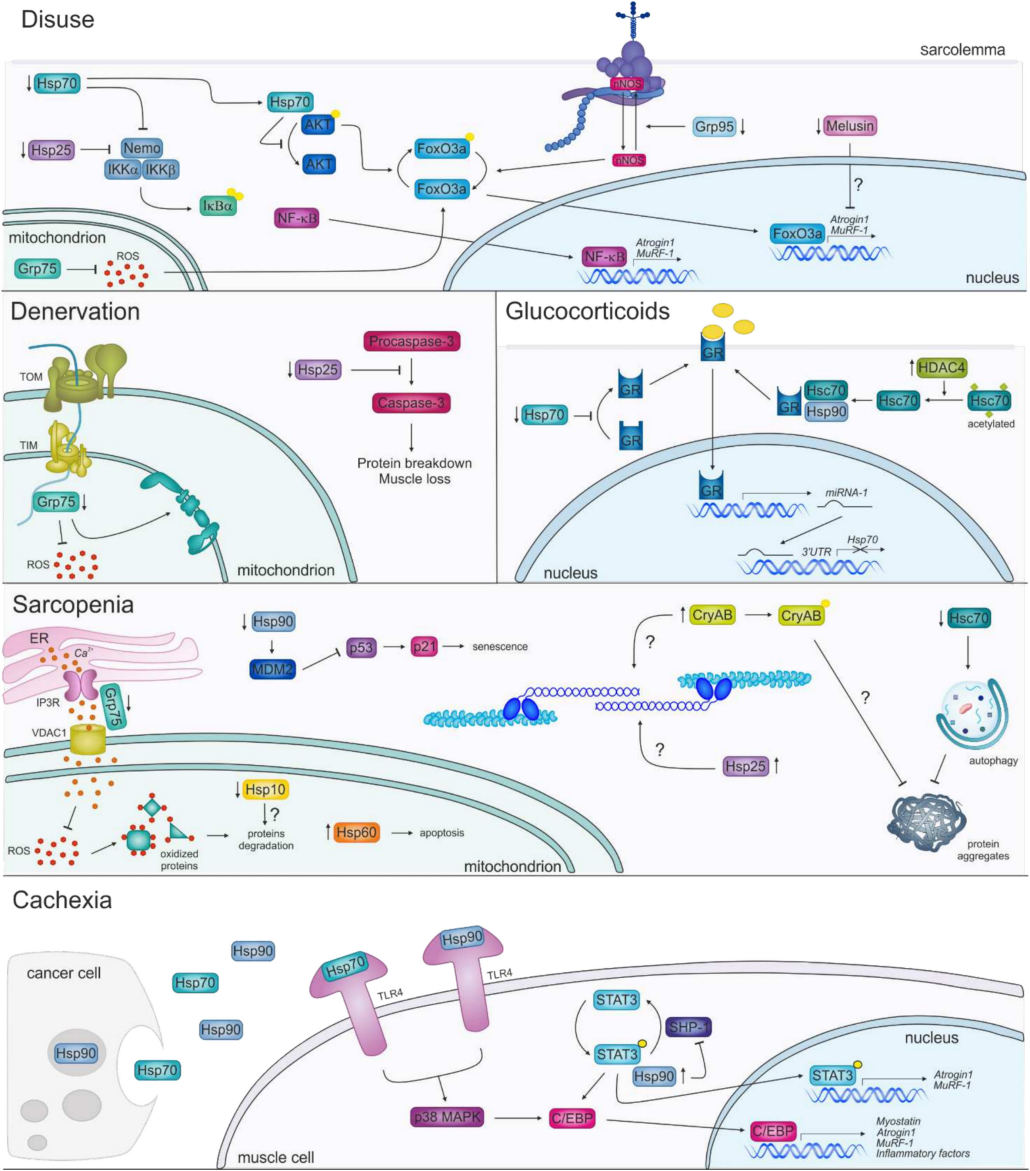
The role of chaperone proteins in muscle wasting. Chaperone proteins have a pivotal role in many processes involved in different type of muscle atrophy. Chaperones regulate crucial intracellular signalling pathways, often leading to the transcription of atrogenes, control sarcomere turnover by refolding unfolded proteins, preventing their aggregation or favouring their degradation through autophagy or the ubiquitin proteasome system. Small arrows next to chaperone proteins indicate up‐ or down‐regulation during muscle wasting. Continuous arrows indicate stimulatory effects, blunt arrows indicate inhibitory effects.

In this review, we summarize the main experimental evidence sustaining the pivotal role of chaperone proteins in skeletal muscle atrophy (Table 2 and Figure 2). For some chaperones, the modulation of their expression during atrophy is still controversial, and there are no conclusive data demonstrating a causative role in atrophy induction. Standardizing experimental and analytical procedures, such as muscle type, time of treatment, age and strain of mice, may allow to obtain more clear and reproducible data. Nonetheless, for chaperones like Hsp70, Grp94, Hsp10, Melusin and Hsp25, experimental data indicate that their reduction in expression is a driving events dictating muscle atrophy onset. Genetic as well as pharmacological strategies demonstrate that blocking this decline rescue muscle wasting or at least significantly ameliorate the phenotype, highlighting chaperone proteins as promising therapeutic targets for muscle atrophy.

## Ethics Statement

The authors have nothing to report.

## Conflicts of Interest

The authors declare no conflicts of interest.

## Supporting information


**Data S1.** Supplementary Information.
