# Supplementary material for: Chaperone Proteins: The Rising Players in Muscle Atrophy
Source: J Cachexia Sarcopenia Muscle. 2024 Dec 20;16(1):e13659. doi: 10.1002/jcsm.13659 (PMC11747685; doi:10.1002/jcsm.13659)
Supplement: Supplementary file 1 — Data S1. Supplementary Information. [file JCSM-16-e13659-s001.docx]

**Chaperone proteins: the rising players in muscle atrophy**

Davide Acquarone^1^, Alessandro Bertero^1^, Mara Brancaccio^1^ and Matteo Sorge^1^

1- Department of Molecular Biotechnology and Health Sciences, University of Turin; Turin; Italy

**Supplementary references**

**S1** M. P. Mayer, "Hsp70 chaperone dynamics and molecular mechanism," *Trends Biochem Sci,* vol. 38, no. 10, pp. 507-14, Oct 2013.

**S2** S. Taurin *et al.*, "Proteome analysis and functional expression identify mortalin as an antiapoptotic gene induced by elevation of [Na+]i/[K+]i ratio in cultured vascular smooth muscle cells," *Circ Res,* vol. 91, no. 10, pp. 915-22, Nov 15 2002.

**S3** A. Hoter, M. E. El-Sabban, and H. Y. Naim, "The HSP90 Family: Structure, Regulation, Function, and Implications in Health and Disease," *Int J Mol Sci,* vol. 19, no. 9, Aug 29 2018.

**S4** S. Song *et al.*, "Luteolin selectively kills STAT3 highly activated gastric cancer cells through enhancing the binding of STAT3 to SHP-1," *Cell Death Dis,* vol. 8, no. 2, p. e2612, Feb 9 2017.

**S5** M. Frasson *et al.*, "Grp94 is Tyr-phosphorylated by Fyn in the lumen of the endoplasmic reticulum and translocates to Golgi in differentiating myoblasts," *Biochim Biophys Acta,* vol. 1793, no. 2, pp. 239-52, Feb 2009.

**S6** A. Altmeyer *et al.*, "Tumor-specific cell surface expression of the-KDEL containing, endoplasmic reticular heat shock protein gp96," *Int J Cancer,* vol. 69, no. 4, pp. 340-9, Aug 22 1996.

**S7** M. Brancaccio *et al.*, "Melusin is a new muscle-specific interactor for beta(1) integrin cytoplasmic domain," *J Biol Chem,* vol. 274, no. 41, pp. 29282-8, Oct 8 1999.

**S8** M. Brancaccio *et al.*, "Melusin, a muscle-specific integrin beta1-interacting protein, is required to prevent cardiac failure in response to chronic pressure overload," *Nat Med,* vol. 9, no. 1, pp. 68-75, Jan 2003.

**S9** S. Vilasi *et al.*, "Chaperonin of Group I: Oligomeric Spectrum and Biochemical and Biological Implications," *Front Mol Biosci,* vol. 4, p. 99, 2017.

**S10** M. Haslbeck, S. Weinkauf, and J. Buchner, "Small heat shock proteins: Simplicity meets complexity," *J Biol Chem,* vol. 294, no. 6, pp. 2121-2132, Feb 8 2019.

**S11** A. Mogk and B. Bukau, "Role of sHsps in organizing cytosolic protein aggregation and disaggregation," *Cell Stress Chaperones,* vol. 22, no. 4, pp. 493-502, Jul 2017.

**S12** W. F. Bluhm, J. L. Martin, R. Mestril, and W. H. Dillmann, "Specific heat shock proteins protect microtubules during simulated ischemia in cardiac myocytes," *Am J Physiol,* vol. 275, no. 6, pp. H2243-9, Dec 1998.

**S13** P. Connell *et al.*, "The co-chaperone CHIP regulates protein triage decisions mediated by heat-shock proteins," *Nat Cell Biol,* vol. 3, no. 1, pp. 93-6, Jan 2001.
